# Supplementary figures and images for: Cannabigerol Induces Autophagic Cell Death by Inhibiting EGFR-RAS Pathways in Human Pancreatic Ductal Adenocarcinoma Cell Lines
Source: Int J Mol Sci. 2024 Feb 7;25(4):2001. doi: 10.3390/ijms25042001 (PMC10888274; doi:10.3390/ijms25042001)

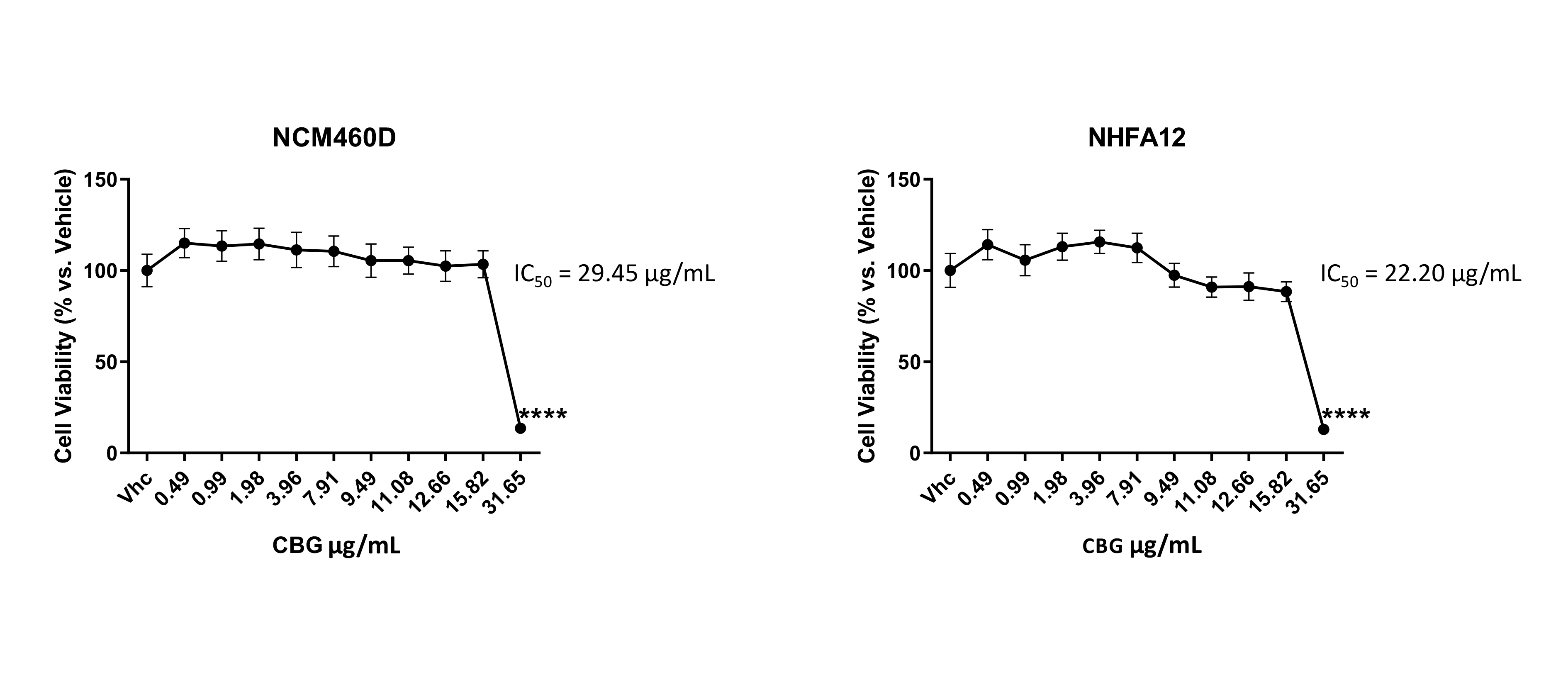

Supplement: Supplementary file 1 [file ijms-25-02001-s001.zip › Suppl Figure 1.tif]
